# Artificial intelligence adoption in healthcare: a systematic review of implementation challenges and health services implications

**DOI:** 10.1186/s12913-026-14960-x

**Published:** 2026-06-13

**Authors:** Giuseppe Lanfranchi, Guido Gembillo, Domenico Santoro, Massimo Villari

**Affiliations:** 1 Department of Mathematics and Computer Sciences, Physical Sciences and Earth Sciences, University of Messina, Messina, Italy; 2 Department of Medicine and Surgery, University of Messina, Messina, Italy

**Keywords:** Artificial intelligence, Technology adoption, Healthcare, Enabling factors, Barriers, Healthcare innovation

## Abstract

The adoption of Artificial Intelligence (AI) in the healthcare sector offers an unprecedented opportunity to revolutionize global health systems by addressing growing challenges related to efficiency, accessibility, and quality of care. This systematic review explores the opportunities, challenges, enablers, and barriers associated with AI adoption in healthcare, integrating professional, organizational, and patient perspectives. Through an in-depth analysis of the literature, this work highlights an integrated set of determinants for AI adoption, including technological, economic, regulatory, and cultural aspects, with particular emphasis on barriers such as perceived threats to professional autonomy, privacy concerns, and infrastructural gaps. As a result, the study develops an evidence-informed integrative framework that explores the interactions between external determinants, such as macroeconomic, technological, and regulatory readiness, and internal factors, including organizational and user readiness. Furthermore, it highlights recurrent knowledge-alignment and coordination challenges between clinical and IT specialists (hereafter referred to as Knowledge-proximity), emphasizing the need for greater integration and mutual understanding to support effective AI implementation. This integrative framework synthesizes multidisciplinary perspectives and provides actionable implications for policymakers, AI providers, and healthcare institutions, with relevance across diverse healthcare contexts and stages of implementation. This research bridges the gap between technological development and real-world implementation, providing a foundation for future studies and evidence-informed strategies to support AI adoption in health services.

**Supplementary Information:**

The online version contains supplementary material available at 10.1186/s12913-026-14960-x.

## Introduction

Artificial Intelligence (AI) is fundamentally reshaping industries across the globe by introducing capabilities for advanced data analysis, sophisticated decision-making support, and automation of routine and complex processes [1]. Industries such as finance are leveraging AI for fraud detection and predictive analytics [2], while retail is utilizing AI for personalized customer experiences and inventory management [3]. Manufacturing is also incorporating AI for predictive maintenance and optimizing production processes [4]. These applications demonstrate the transformative impact of AI across diverse domains, highlighting its potential to revolutionize operations and decision-making at an unprecedented scale [5]. In the healthcare domain, AI’s potential is particularly revolutionary, offering improvements in areas such as diagnostics, personalized treatment pathways, operational efficiency, and patient outcomes [6]. Technologies like Machine Learning (ML) and Natural Language Processing (NLP) are leading this transformation, enabling the extraction of valuable insights from both structured data, such as medical imaging, and unstructured data, including clinical notes and research papers [7, 8]. These technological advancements are providing a powerful toolkit for addressing some of healthcare’s most urgent challenges, such as limited resources, increasing patient volumes, rising costs, and the need for enhanced care quality [9]. Despite its vast potential, integrating AI into healthcare systems is not without its hurdles, many of which are uniquely challenging in this sector. Ethical concerns tied to patient confidentiality are particularly acute in healthcare, where sensitive personal data is at the core of operations [10]. Unlike other industries, healthcare must also navigate complex regulatory landscapes designed to protect patient safety and privacy, which can slow the adoption of innovative technologies [11]. Additionally, healthcare professionals often perceive AI as a potential threat to their clinical autonomy, creating resistance that is less prevalent in other sectors [12]. Infrastructure inadequacies, such as outdated informatic systems that cannot easily integrate advanced technologies, further compound these challenges [13]. Therefore, the successful deployment of AI in healthcare depends not only on technological readiness but also on the capacity of organizations to adapt and on fostering the willingness of individual stakeholders to embrace innovation [14]. This intricate scenario highlights the uniquely multifaceted nature of the adoption process in healthcare, underscoring the need for tailored strategies to address its specific complexities [15].

The purpose of this systematic review is to synthesize and critically organize existing evidence on the factors influencing AI adoption and implementation in healthcare settings, with particular attention to implications for health services organization and management. While prior reviews have examined organizational readiness or healthcare professionals’ attitudes toward AI adoption [16, 17], they tend to address determinants at specific levels of analysis. Less attention has been devoted to how technological, organizational, and professional dimensions interact during implementation, particularly in relation to cross-professional knowledge alignment between clinical and technical actors. Building on this stream, our review advances a health services perspective by jointly organizing internal and external determinants of AI adoption. We conceptualize knowledge-proximity as a cross-level coordination mechanism capturing recurrent Knowledge-alignment challenges between clinical and IT professionals, namely differences in expertise, language, and professional logics that shape implementation processes and condition technology acceptance. Specifically, this review aims to answer the following research questions (RQs):

### RQ1

What are the internal and external factors influencing AI adoption in healthcare?

### RQ2

How do coordination and knowledge-alignment challenges between clinical and IT specialists shape AI implementation processes in healthcare settings?

**Fig. 1:**
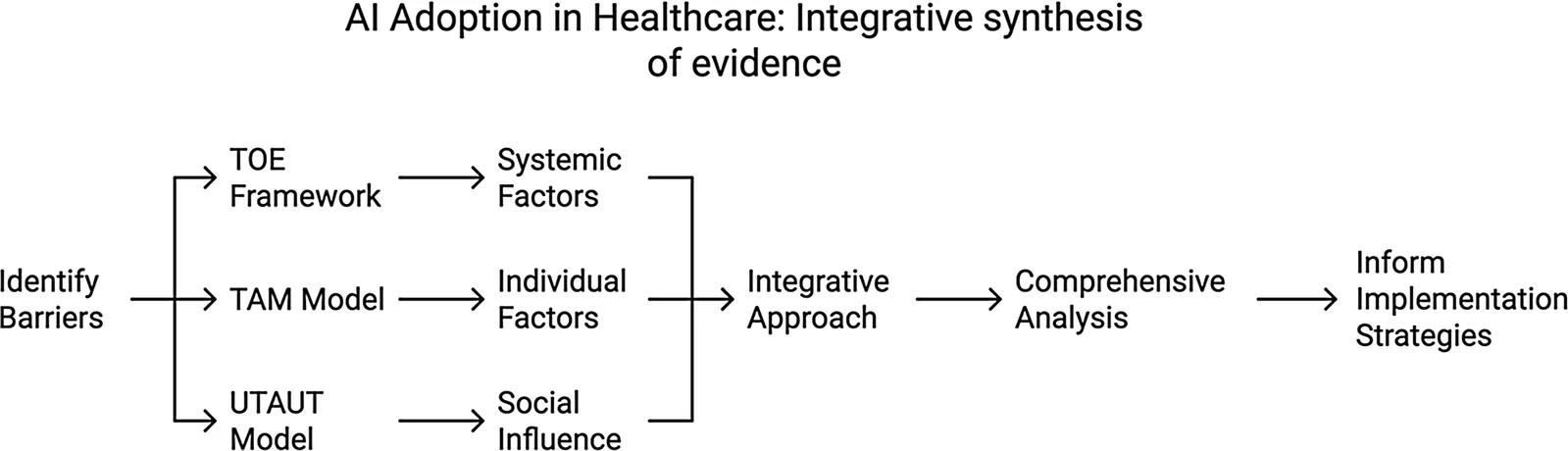
Integrative synthesis of factors influencing AI adoption in healthcare

This review advances prior work in three main ways. First, it provides an integrated health services synthesis that jointly organizes internal and external determinants of AI adoption across technological, organizational, and environmental dimensions. Second, it introduces knowledge-proximity as an interpretive mechanism linking individual acceptance dynamics within the Technology Acceptance Model [18] to organizational readiness processes captured by the Technology–Organization–Environment (TOE) framework [19], thereby clarifying how cross-professional alignment shapes implementation outcomes. Third, it moves beyond descriptive categorization by identifying cross-contextual adoption patterns, highlighting how institutional configuration, AI domain specificity, and digital maturity condition deployment pathways. By synthesizing existing literature using a PRISMA-guided systematic approach, the review identifies pathways for overcoming barriers and maximizing the benefits of AI integration in health services contexts. Grounded in the TOE framework, the review draws on established adoption lenses as organizing frameworks to analyze how technological, organizational, and environmental factors interact to facilitate or hinder AI adoption in healthcare. A systematic methodology was employed to identify, evaluate, and synthesize relevant studies, ensuring rigor and transparency for meaningful insights [20]. The resulting integrative framework highlights the importance of aligning organizational priorities, technological capabilities, and implementation practices, including cross-professional collaboration (Fig. 1). By addressing these challenges, the framework supports a more structured understanding of AI implementation in healthcare services rather than proposing a new theoretical model. Moreover, by tackling communication and knowledge-alignment issues that disrupt implementation processes, the framework underscores the importance of fostering mutual understanding and teamwork across professional groups. By shedding light on both the enablers and barriers to AI adoption, this review aims to inform health services decision-making and guide future empirical research.

The findings have the potential to shape future research by identifying gaps that require further exploration, such as the role of interdisciplinary collaboration in enhancing adoption. Additionally, they offer practical guidance for implementing tailored strategies that address sector-specific challenges, paving the way for more effective and scalable AI integration. To structure this exploration, the review is organized into several sections: Section “Theoretical background” outlines the theoretical framework; Section “Methodology” explains the methodology; Section “Results” analyses results and discussion, presenting key findings; and Section “Discussion” involves the conclusion, where actionable recommendations and implications are highlighted.

## Theoretical background

### Research setting

The composition of the healthcare industry faces challenges such as disparities related to workforce shortages, resource allocation, and technology adoption [21]. Prior research consistently documents how difficulties in recruiting qualified personnel, exacerbated by an aging population, create imbalances between the demand for and supply of healthcare services. According to [22], a projected shortage of 18 million healthcare workers is expected by 2030. Compounding this issue are inefficient and often complex workflows that delay the delivery of care, increase wait times, and compromise service quality. Importantly, these challenges manifest differently across healthcare systems, and vary significantly across low-, middle-, and high-income countries. For example, workforce shortages are particularly pronounced in low- and middle-income nations, further compounded by limited access to technological infrastructure, which exacerbates inequalities in care delivery [23]. Conversely, high-income countries face mounting hospital pressures driven by an aging population and the prevalence of chronic diseases, which strain already burdened healthcare systems. Chronic diseases are also a major contributor to rising healthcare costs, which are further inflated by the need to maintain cutting-edge infrastructure. These financial pressures deepen regional disparities, as not all regions have the necessary resources to ensure equitable access to quality care. Such heterogeneity highlights the fragmented nature of healthcare systems and calls for tailored approaches that account for regional needs and infrastructural variations [24]. In this context, the implementation of advanced digital technologies, including AI, is shaped by system-level constraints and contextual factors, requiring adaptation to specific organizational and institutional settings to achieve meaningful impact [25].

### The opportunity of AI

In this context, AI emerges as a transformative technology with the potential to address these diverse challenges by improving operational efficiency, alleviating workloads, and optimizing resource allocation across various healthcare settings [26]. According to a report by Accenture [27], the global AI healthcare market was valued at approximately $6.7 billion in 2021 and is projected to grow at a compound annual growth rate (CAGR) of 41.7% through 2028, indicating strong institutional and market interest in AI-enabled solutions within healthcare systems. This growth is driven by AI’s capacity to support a wide range of applications, including diagnostics, personalized treatment plans, operational enhancements, and the optimization of patient care across different levels of healthcare delivery.

The driving force behind these advancements lies in enabling technologies such as ML and NLP, which significantly enhance the analysis and interpretation of large volumes of structured and unstructured data, such as medical imaging, clinical notes, and genetic information [7, 8]. Empirical studies have reported promising performance levels in specific clinical applications; for instance, Abdalla et al. [28] showed that AI-powered tools achieved accuracy rates of up to 90% in detecting conditions such as diabetic retinopathy, under controlled conditions and specific use cases. AI’s potential is also evident in predictive analytics, where algorithms are used to anticipate patient outcomes and support resource planning and allocation decisions [29]. In addition, applications in robotics, including surgical assistance and automated medication dispensing, have demonstrated the potential to reduce recovery times, decrease post-operative complications, and enhance operational efficiency in hospital environments [30]. Collectively, these applications illustrate the breadth of AI use cases in healthcare and their relevance for improving service delivery, rather than guaranteeing uniform performance across contexts. Table 1 provides a summary of AI’s key applications in the healthcare sector.

**Table 1:** Main application of AI technologies in healthcare

Application Area	Description	Examples
Robotics	AI-powered robots assist in surgeries, automate tasks, and support elderly care.	Surgical robots (e.g., da Vinci), elder care robots.
Diagnostics	AI aids in early detection of diseases like cancer and diabetic retinopathy with high accuracy.	AI diagnostic tools for breast cancer, retinal disease detection.
Predictive Analytics	Algorithms forecast patient outcomes, predict disease outbreaks, and optimize resources.	COVID-19 outbreak prediction, resource allocation algorithms.
Clinical Decision Support Systems (CDSS)	AI provides clinicians with data-driven recommendations for diagnoses and treatments.	Systems for triage in emergency departments.
Personalized Medicine	Tailors treatments based on patient genetic profiles and medical history.	Genomic analysis for precision treatments.
Medical Imaging	AI analyzes imaging data, such as X-rays and MRIs, to identify abnormalities.	AI in radiology for identifying tumors.
Telemedicine	Enables remote consultations and patient monitoring through AI-powered platforms.	Remote monitoring for chronic diseases.
Drug Discovery	Accelerates drug development by predicting molecular interactions and identifying candidates.	AI platforms for drug repurposing and new molecule discovery.
Operational Efficiency	Optimizes hospital workflows, manages resources, and reduces administrative burdens.	Automated scheduling, inventory management systems.
Health Monitoring and Prevention	AI systems for continuous monitoring of vital signs and early detection of health issues.	Wearables for heart rate and oxygen monitoring (e.g., Apple Watch, Fitbit).
Digital Therapeutics	Applications that support treatments for chronic or mental health conditions using AI.	CBT apps for depression and anxiety (e.g., Woebot, Wysa).
Rare Disease Management	AI for identifying rare diseases through genetic and phenotypic data analysis.	Platforms like Face2Gene for identifying genetic syndromes.
AI in Pathology	AI supports pathologists in analyzing biological tissues, expediting diagnoses.	AI tools for analyzing biopsies to detect cancer cells with high accuracy.

Unfortunately, the adoption of AI presents several critical barriers to its widespread implementation in the healthcare sector. The literature employs various models to analyze this adoption process, such as the TOE, the TAM, and the Unified Theory of Acceptance and Use of Technology (U-TAUT) [31]. These frameworks are widely used in healthcare and information systems research to examine adoption dynamics at different analytical levels.

The TOE framework [19] considers available technology, organizational readiness, and environmental conditions as key determinants of organizational adoption. Alongside the TOE, the TAM [18] focuses on individual-level factors influencing acceptance, particularly perceived usefulness and ease of use. Similarly, U-TAUT emphasizes the role of social influence, facilitating conditions, and performance expectations in shaping technology use within organizational settings [31].

In this review, these frameworks are used as complementary organizing lenses to structure and interpret prior evidence on AI adoption in healthcare. This multilevel perspective reflects the heterogeneous nature of healthcare systems, where adoption processes are shaped by interactions between system-level constraints, organizational capabilities, and individual perceptions. Accordingly, the review leverages these frameworks to categorize barriers and enablers reported in the literature, rather than to advance or extend adoption theory.

However, while TAM explains how individual perceptions of usefulness and ease of use shape technology acceptance, and TOE captures how technological, organizational, and environmental conditions influence adoption decisions, neither framework explicitly addresses the epistemic distance that may exist between professional groups involved in AI development and use. In healthcare contexts, clinicians and IT specialists operate within distinct knowledge domains, evaluative criteria, and decision logics. Such cognitive and operational distance can hinder the translation of technological potential into clinically meaningful practice, even when institutional readiness and technological capabilities are present. To clarify this gap, the review introduces the notion of Knowledge-proximity as a cross-level coordination mechanism linking individual acceptance and organizational preparedness. In doing so, it offers a more integrated interpretation of how micro-level perceptions and macro-level structures interact during AI implementation processes.

## Methodology

Our analysis was conducted in January 2025, following a systematic review process guided by the PRISMA-guided systematic review protocol (Preferred Reporting Items for Systematic Reviews and Meta-Analyses) in accordance with PRISMA 2020 recommendations [32] (Appendix A). The review followed the standard PRISMA stages of identification, screening, eligibility assessment, and inclusion.

The bibliometric component was used to systematically map the structure and evolution of the literature, while the qualitative synthesis was employed to interpret adoption-related themes and implementation challenges. Bibliometric indicators were extracted using the open-source R package bibliometrix [33, 34]. Bibliometric analysis was used as a complementary analytical tool to enhance transparency and rigor, rather than as a substitute for qualitative interpretation. The bibliometric analysis enabled the examination of publication trends, citation patterns, co-authorship networks, and keyword co-occurrence structures, supporting an initial structuring of the evidence base. Considering our research questions, we leveraged the Scopus database to retrieve records relevant to AI adoption in healthcare. Scopus was selected due to its multidisciplinary scope and comprehensive coverage of peer-reviewed journals across medicine, health professions, management, and information systems research [35, 36]. This database is widely used in bibliometric and systematic reviews due to its consistent indexing standards and breadth of journal coverage. The final database search was conducted in January 2025.

**Fig. 2:**
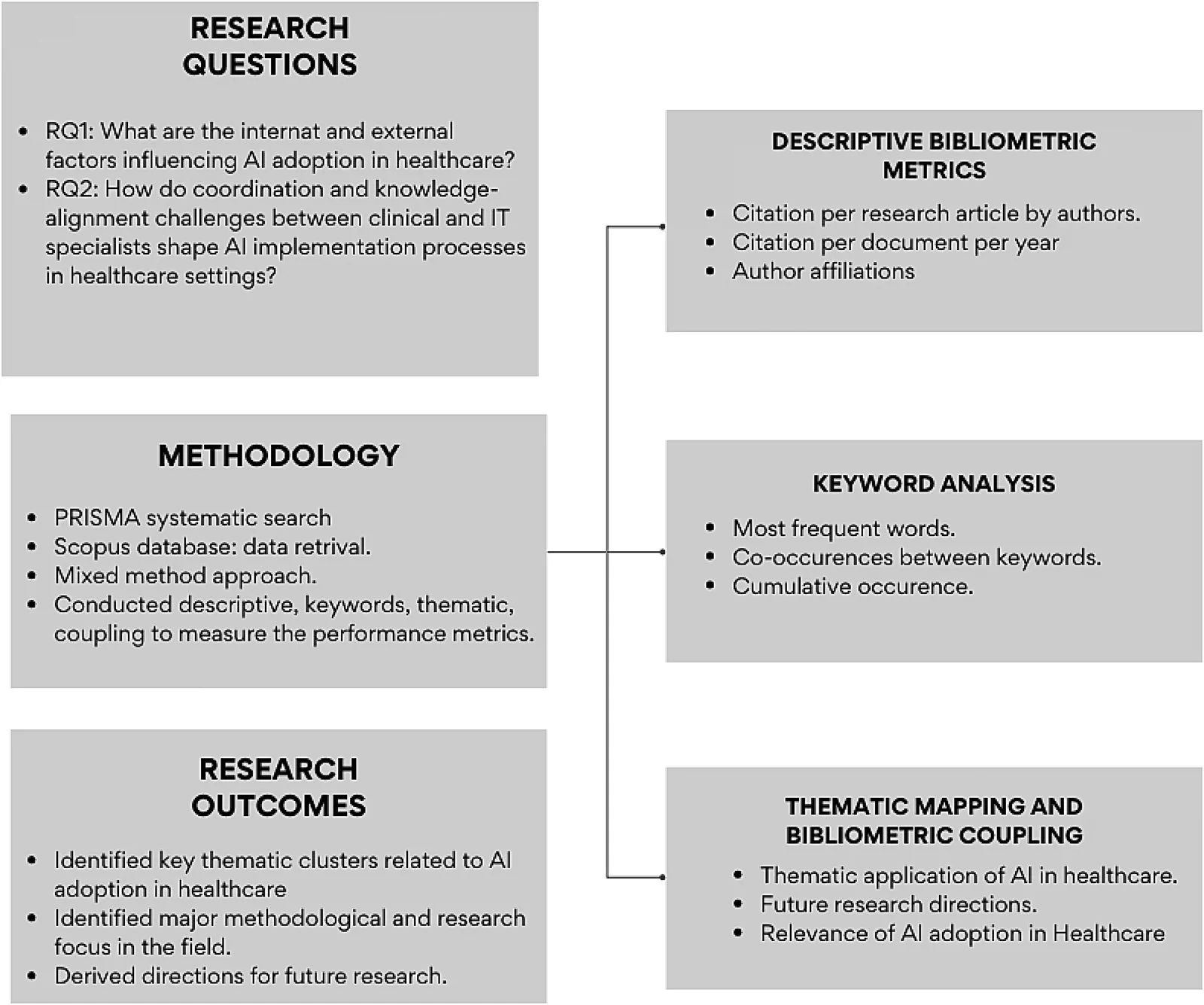
Overview of the systematic review design

The review process was structured in four sequential phases (Fig. 2). The first phase involved the definition of the review objectives and research questions, followed by the development of the search strategy and eligibility criteria.

Inclusion criteria comprised peer-reviewed journal articles focusing on the adoption, implementation, or organizational implications of AI in healthcare settings. Studies were required to address managerial, professional, organizational, or policy-related aspects of AI adoption. Exclusion criteria included studies exclusively focused on algorithm development, technical performance evaluation, or experimental validation without implications for healthcare organization, adoption, or implementation processes.

The second phase consisted of a systematic database search and initial screening, conducted in line with PRISMA recommendations to ensure transparency and replicability. Duplicate records were identified and removed prior to screening. Titles and abstracts were then screened to exclude studies clearly unrelated to AI adoption and health services research.

The third phase involved full-text eligibility assessment, during which articles were evaluated against predefined inclusion and exclusion criteria. Studies were excluded if they focused exclusively on technical model development, algorithmic performance testing, or experimental clinical validation without organizational or adoption-related implications. Qualitative coding and thematic synthesis were conducted by the four authors. After jointly defining an initial coding framework, the authors independently coded the included studies. Coding decisions were compared through iterative discussions, and discrepancies were resolved through consensus, leading to the final set of themes [35].

In addition to thematic outcomes, data were extracted on study characteristics and contextual variables, including publication year, country or geographical context, healthcare setting, AI application domain, methodological approach, and the type of organizational actors involved. Where information was unclear or not explicitly reported in the included studies, no assumptions were made and the data were coded as not specified. A formal risk of bias assessment was not conducted, as the included studies were not evaluated for clinical effectiveness or comparative intervention outcomes. Consistent with the qualitative and organizational focus of the review, the analysis aimed to synthesize adoption-related themes and implementation challenges rather than to assess methodological quality or internal validity using standardized risk of bias tools. All studies included after title/abstract screening and full-text eligibility assessment were retained for both the bibliometric analysis and the qualitative thematic synthesis. No data transformations or quantitative preparations were required prior to synthesis. Results were presented using descriptive tables and figures derived from bibliometric and qualitative analyses. Potential heterogeneity and sensistivity analyses among studies were not explored given the qualitative and interpretive nature of the synthesis.

Based on this framework, comprehensive search strategy was developed combining relevant terms to identify pertinent articles. Employing an inductive approach, we crafted a detailed query encompassing various facets of AI and healthcare. The query used is as follows: TITLE-ABS-KEY ((“Artificial Intelligence” OR “AI” OR “Machine Learning” OR “Deep Learning” OR “Natural Language Processing” OR “Computational Intelligence”) AND (“Healthcare” OR “Hospital*” OR “Clinic*” OR “Medical Sector” OR “Health Care Sector”) AND (“Adoption” OR “Implementation” OR “Utilization” OR “Acceptance” OR “Deployment” OR “Integration”) AND (“Management” OR “Organizational Factors” OR “Governance” OR “Decision-Making” OR “Hospital Administration” OR “Policy”)).

This search yielded 10,878 records. Following duplicate removal (*n* = 214), 10,664 records were screened at title and abstract level. To ensure relevance and timeliness, records were restricted to English-language publications published between 2014 and 2024, resulting in 5,047 records.

Subsequently, disciplinary filters were applied to retain studies published in medicine, health professions, nursing, computer science, engineering, decision sciences, business and management, social sciences, and multidisciplinary journals, reducing the dataset to 4,716 records. To enhance comparability across heterogeneous study designs and ensure a minimum level of reporting transparency in an interdisciplinary corpus, we retained only articles published in Scimago Journal Rank (SJR) Q1 journals. This criterion was adopted as a pragmatic proxy for editorial and methodological consistency, supporting a more coherent qualitative synthesis, while acknowledging the potential exclusion of relevant contributions published in lower-ranked or specialized outlets. This step yielded 999 articles across 52 journals [36].

Following title and abstract screening, 122 articles were selected for full-text assessment. After eligibility evaluation, studies focusing narrowly on technical or experimental AI applications without organizational or adoption relevance were excluded. The final sample consisted of 75 articles, which formed the basis for both the bibliometric analysis and the qualitative synthesis.

## Results

### Descriptive analysis

Table 2 encapsulates key quantitative insights derived from the selected dataset. The final corpus consists of 75 papers, exhibiting an average citation count of 22.49 between 2017 and 2024. Notably, while the original search encompassed publications from 2014 to 2024, the refinement process filtered out older works, with 2017 emerging as the earliest publication year in the final selection. This indicates that research on AI adoption in healthcare has intensified in more recent years, reflecting the evolving nature of the field. These papers span 21 distinct journals, highlighting the multidisciplinary character of the literature and the dispersion of contributions across different publication outlets.

Authorship trends reveal contributions from 574 researchers, resulting in an average of 7.78 co-authors per article. Only one paper was authored by a single individual, underscoring the collaborative nature of research on AI adoption in healthcare. In addition, international collaboration is evident, as 44.59% of papers involve cross-border co-authorships. This suggests that the study of AI adoption in healthcare is characterized by a globally distributed research community, reflecting shared challenges and interests across healthcare systems.

**Table 2:** Main informations about data, authors and collaboration between authors

Description	Results
***Main informations about data***	
Timespan	2017–2024
Sources (Journals, books, etc.)	21
Documents	75
Annual Growth Rate %	65.49%
Document Average Age	2.27
Average Citations per Document	22.49
References	4836
***Authors***	
No. of Authors	574
Authors keywords	305
Authors of single-authored documents	1
***Authors collaboration***	
Co-authored documents	7.78
International co-authorships %	44.59

**Fig. 3:**
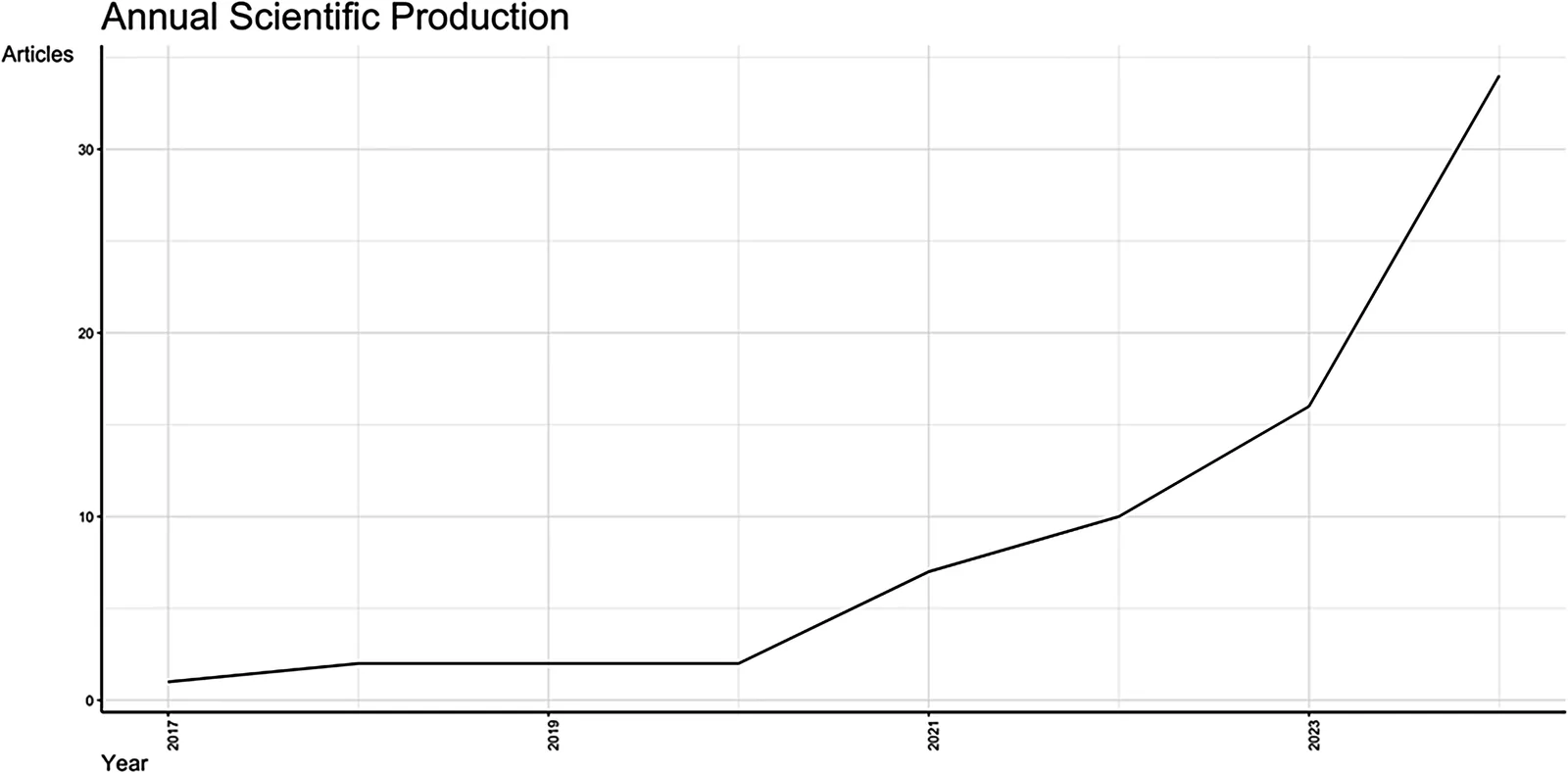
Annual scientific production

Figure 3 presents a temporal overview of the annual scientific output concerning AI adoption in healthcare, showing a clear upward trend over time. The data indicate an annual growth rate of 65.49%, reflecting a substantial increase in scholarly attention to the topic. This trend is consistent with the broader expansion of research on digital technologies in healthcare systems. While publication activity increased gradually in earlier years, a marked rise is observed after 2020, followed by a further acceleration from 2023 onward, culminating in the highest number of publications in 2024. Overall, the pattern suggests growing academic engagement with AI adoption in healthcare, in parallel with ongoing technological and organizational developments in the sector.

**Fig. 4:**
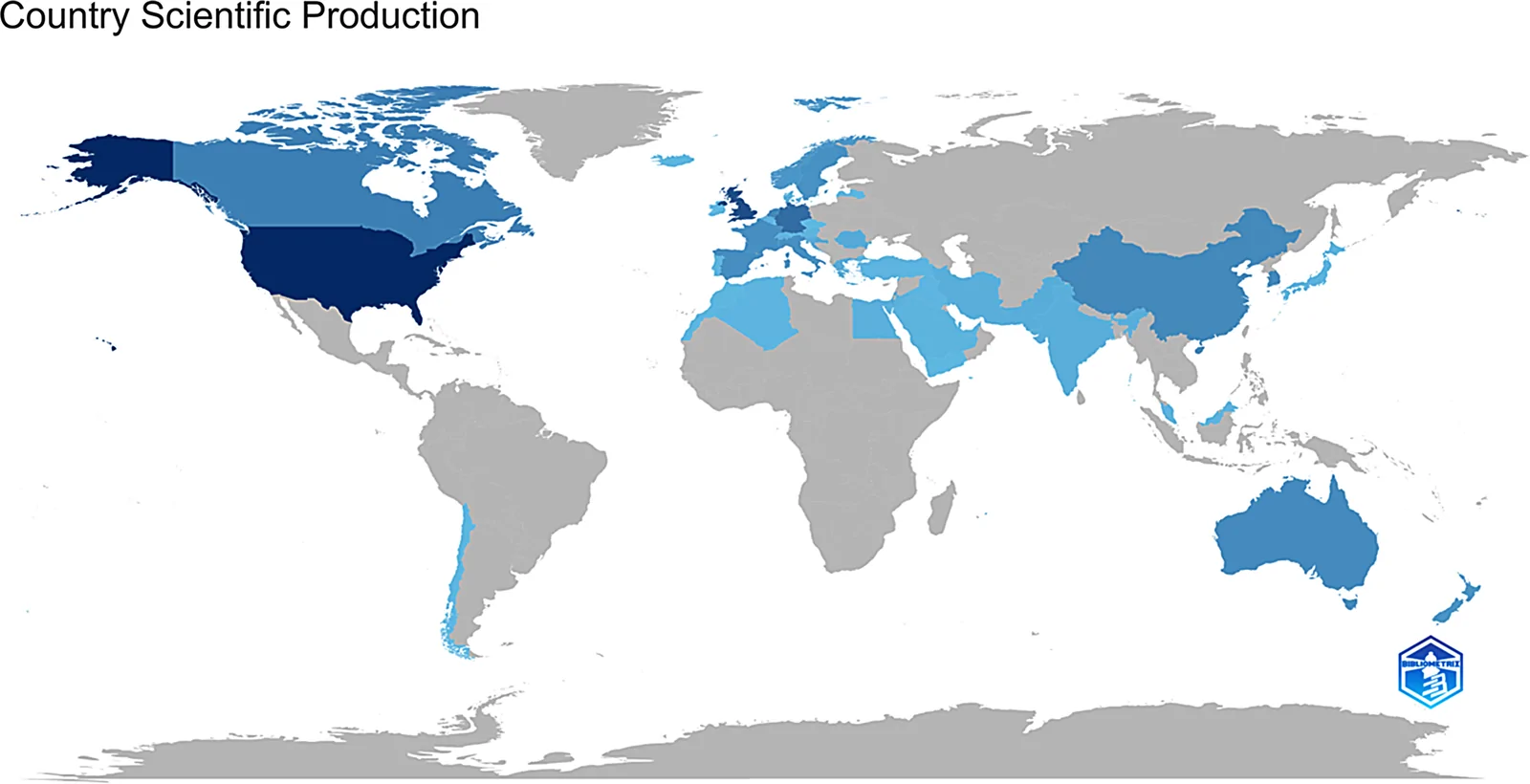
Country scientific production

At the country level, the analysis shows that research on AI adoption in healthcare is distributed across multiple national contexts, reflecting a broad international interest in the topic. As depicted in Fig. 4, the USA and the UK emerge as the most active contributors in terms of publication volume. These countries account for a substantial share of the literature, consistent with their established research infrastructures and longstanding engagement with digital health and AI-related innovation. Differences in publication output across countries indicate an uneven distribution of research activity, with higher concentrations observed in a limited number of national contexts. Beyond these leading contributors, several other countries display notable research activity. Germany, Canada, and China are among the most represented, reflecting their sustained investments in technological development and healthcare research. In addition, South Korea shows a growing presence in the literature, suggesting increasing scholarly engagement with AI adoption in healthcare. This trend is consistent with the country’s broader technological capabilities, including strengths in digital and AI-related industries, as also reflected in Table 3.

Shifting focus to institutional output, the analysis highlights variation in publication activity across organizations. The University of Liverpool (UK) appears as the most prolific institution, with 12 publications, contributing to the UK’s overall prominence in this research area. London South Bank University (UK) also features prominently, indicating a concentration of research activity within specific academic institutions in the UK.

Among US-based organizations, the Stanford University School of Medicine is a leading contributor. Notably, the dataset also includes contributions from non-academic organizations, such as Holmusk Technologies (USA), a private company active in digital and behavioral health, with nine publications related to AI adoption in healthcare. This finding illustrates the participation of industry actors alongside academic institutions in producing research on AI adoption, pointing to a diversified research landscape involving multiple types of organizations.

**Table 3:** The top 10 productive countries and citations per country

Country	Total Freq	Average article citations
USA	93	24,9
UK	62	136
Germany	41	16,3
Canada	30	14,8
China	25	48
South Korea	25	17,7
Netherlands	24	37,8
Australia	23	6,3
France	21	20
Spain	21	15,7

**Fig. 5:**
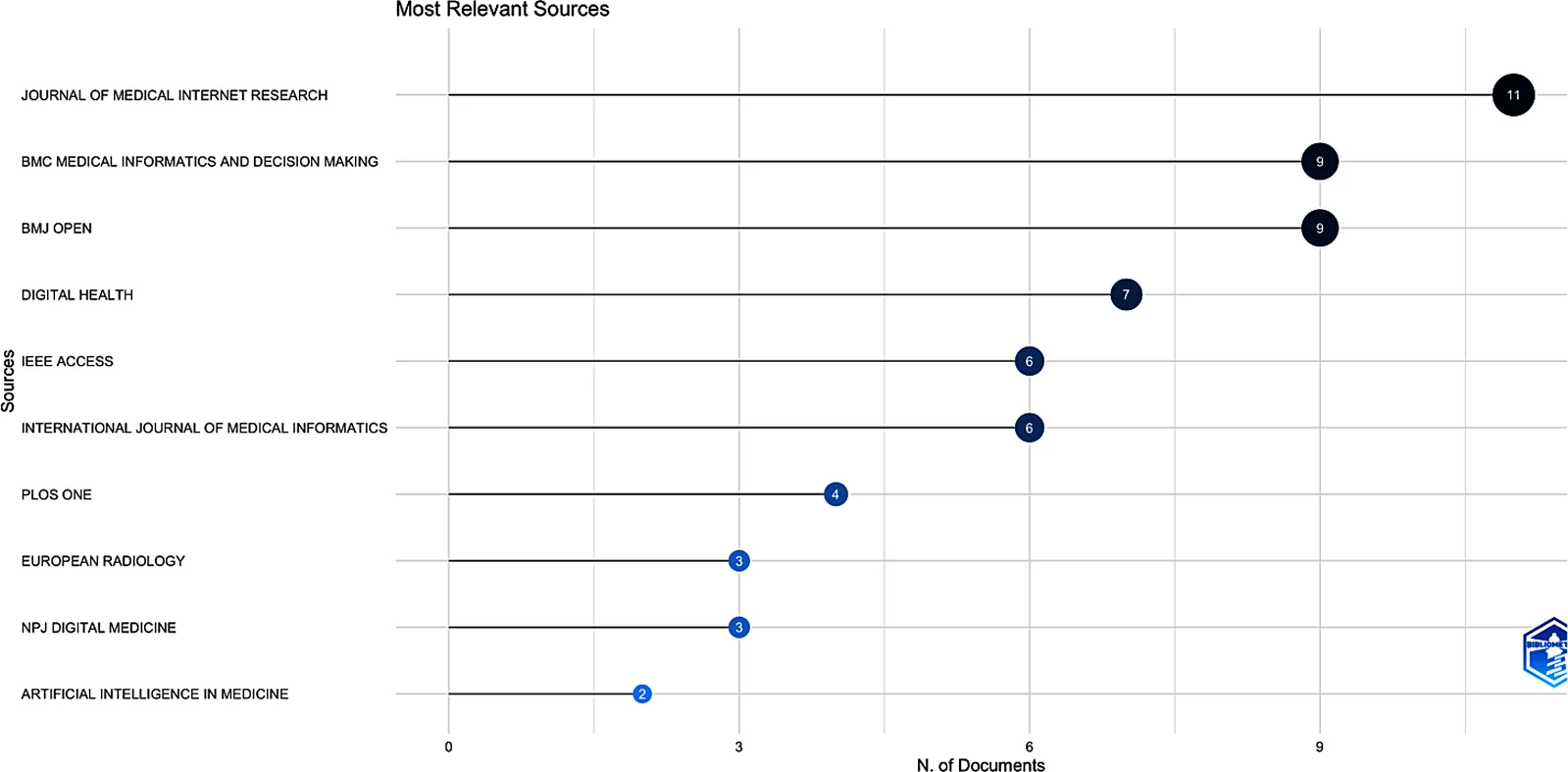
Most relevant journals

The analysis of the most productive publication sources, illustrated in Fig. 5, highlights the 10 most prolific journals in the dataset. No pronounced concentration is observed among the top outlets, as publication volume appears relatively distributed across journals, indicating engagement with the topic across multiple publication venues. Leading the list is the Journal of Medical Internet Research, with 11 published articles, followed by BMC Medical Informatics and Decision Making, with nine publications. These journals are among the most represented outlets in the dataset, reflecting their established focus on digital health, medical informatics, and technology-enabled healthcare research. Other journals contribute smaller but comparable numbers of publications, suggesting that research on AI adoption in healthcare is not confined to a single dominant outlet.

Overall, the distribution of publication sources indicates that AI adoption in healthcare is addressed across a range of journals concerned with medical informatics, health information technology (IT), and healthcare systems. The presence of multiple journals with similar publication volumes points to a diversified publication landscape, in which contributions are disseminated across different editorial domains.

### Content analysis

To map the primary research trajectories, identify existing gaps, and outline future directions for AI adoption in healthcare, this study employs a content analysis approach. By examining yearly topic trends, the analysis shows changes in the prominence of AI-related themes over time. In particular, AI-related topics reach a peak in 2023, followed by a diversification of research themes in 2024, with attention shifting toward closely related application domains.

One of the most prominent emerging topics concerns electronic health records (EHRs), which appear increasingly frequently in recent publications. The literature indicates that AI is being explored in relation to how clinical data are structured, accessed, and used within healthcare organizations. Studies addressing AI-enabled EHRs often focus on issues such as data management, interoperability, and administrative processes, rather than on technical development alone.

Another recurring thematic area relates to clinical decision-making, where research increasingly examines the use of AI to support clinical tasks. This includes applications related to diagnostic support and treatment recommendations, with studies reporting on potential implications for clinical workflows and decision processes.

**Fig. 6:**
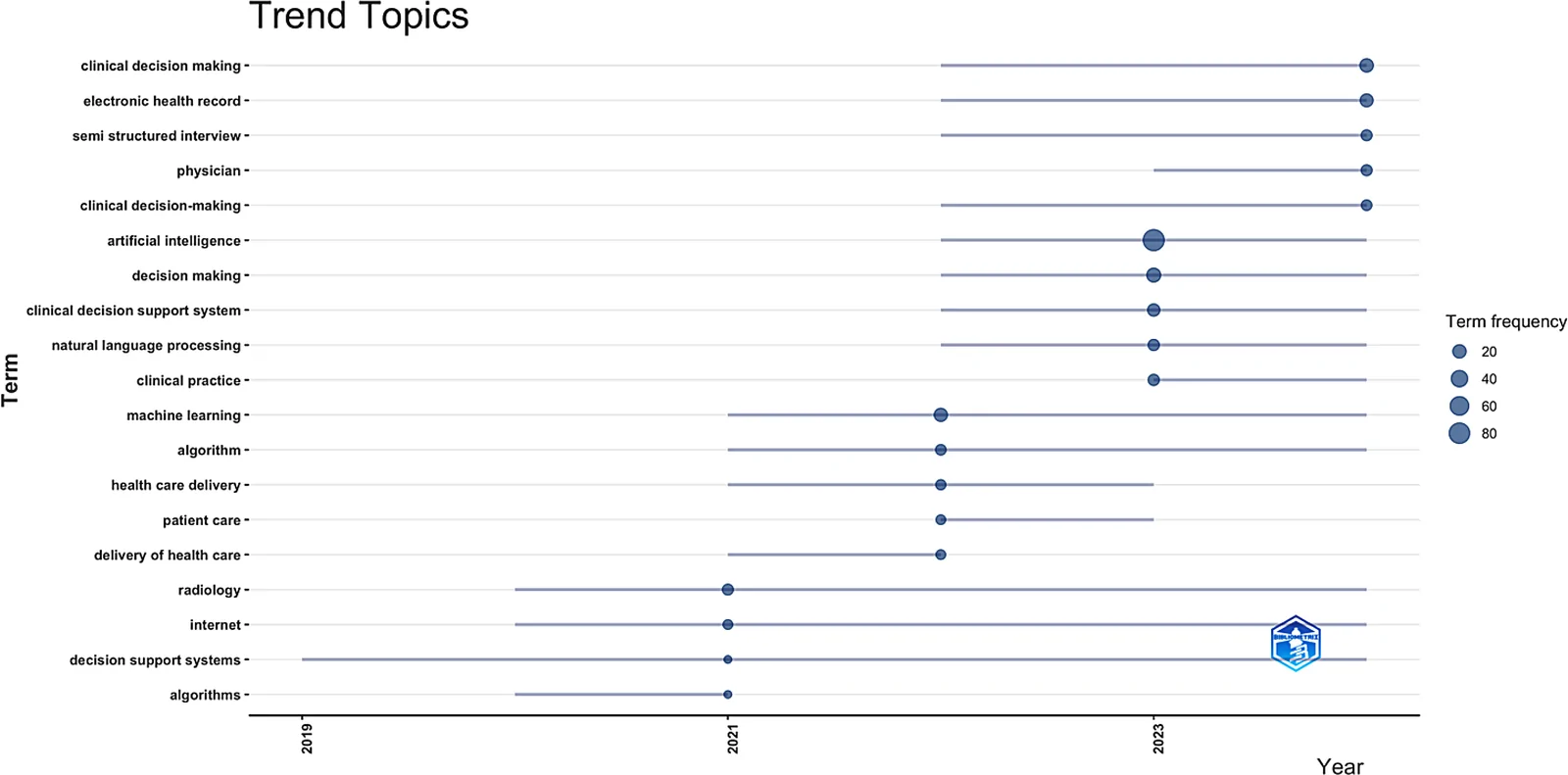
Trend topics

Looking at earlier years, prior research emphasized ML methods and algorithmic approaches applied to healthcare contexts. These contributions form the background against which more recent studies have shifted attention toward applied and organizational aspects of AI use, reflecting a gradual transition from methodological development to implementation-oriented research. In sum, the observed thematic evolution highlights a growing emphasis on applied AI use cases in healthcare, particularly in relation to EHR management and clinical decision support. Figure 6 presents the 16 most frequently occurring research topics by year, providing an overview of how thematic priorities have evolved over time.

**Fig. 7:**
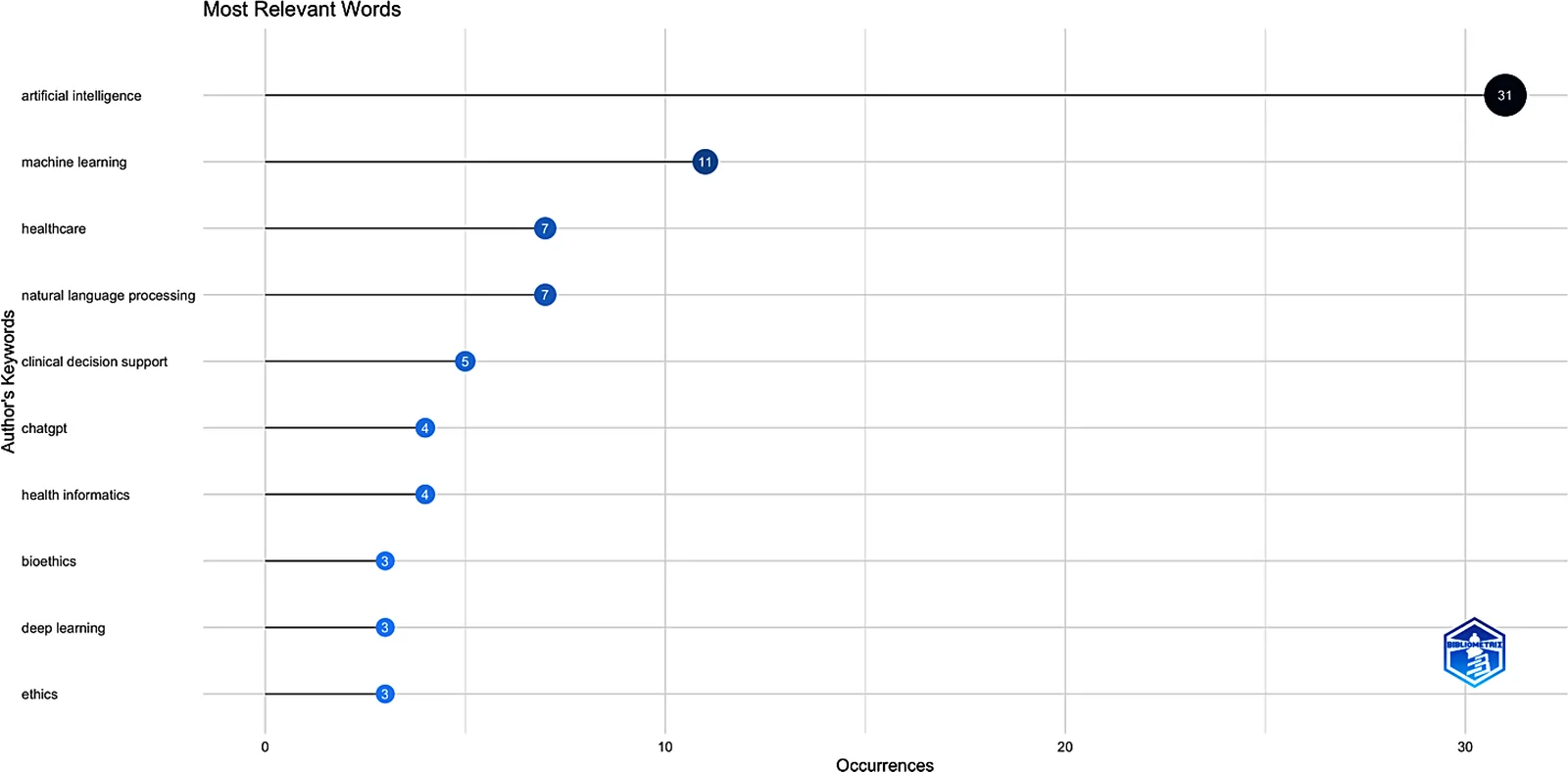
Most relevant keywords

Expanding upon the previous analysis, the bar chart in Fig. 7 provides a quantitative overview of the most frequently occurring keywords within the research corpus, highlighting dominant thematic areas. The term “artificial intelligence” appears most frequently, reflecting its central role in the literature. This is followed by “healthcare” and “machine learning (ML)”, indicating a strong focus on AI-related methods and applications within healthcare contexts. Keywords such as “clinical decision support” and “natural language processing (NLP)” also feature prominently, suggesting increasing attention to AI applications supporting clinical decision-making and the analysis of unstructured clinical data. In addition, the presence of “ChatGPT” indicates emerging scholarly interest in large language models (LLMs), particularly in relation to clinical documentation, information retrieval, and patient-facing applications. Beyond technical terms, keywords related to health practice and bioethics are also represented, pointing to parallel discussions on the organizational, ethical, and governance implications of AI adoption in healthcare. These themes reflect concerns related to data protection, patient privacy, and ethical decision-making, which are frequently discussed alongside technological developments. Furthermore, the occurrence of “deep learning” and “ethics” highlights the co-presence of methodological and normative considerations within the literature. Overall, the keyword distribution suggests that research on AI adoption in healthcare addresses both technological advances and broader ethical and organizational issues, as reflected in the thematic composition of the corpus.

### Thematic analysis

**Fig. 8:**
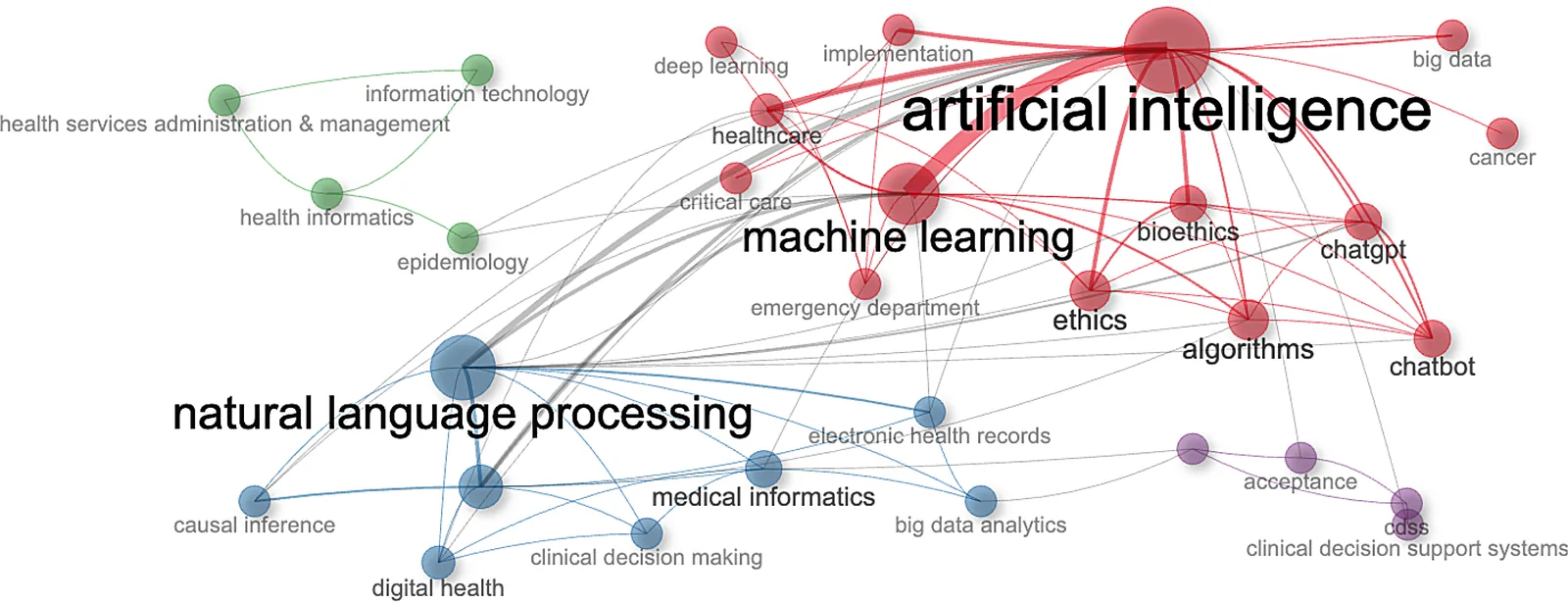
Thematic clustering

The conceptual structure presented in Fig. 8 provides a thematic breakdown of the most interconnected research topics, offering a visual representation of how themes related to AI adoption in healthcare are associated within the literature. The node size reflects term prominence, while the edges illustrate semantic relationships, indicating the co-occurrence of concepts across studies. At the center of the network, AI appears as the most prominent term, reflecting its central position in the research corpus. Closely connected to AI, ML emerges as a major associated domain, linked to themes such as ethics, bioethics, and algorithms, indicating the coexistence of methodological and ethical considerations within the literature.

Terms such as “ChatGPT” and “chatbot” appear in proximity to bioethics and healthcare, suggesting that conversational AI and large language models are increasingly discussed in relation to ethical, organizational, and clinical considerations. These associations point to growing scholarly attention to issues such as reliability, interaction with patients, and responsible use of AI technologies, rather than implying direct clinical impact.

Another distinct cluster centers on NLP, which is closely associated with medical informatics, digital health, and clinical decision-making. This pattern reflects the frequent discussion of NLP in relation to the processing and management of clinical text data within healthcare systems.

In addition, a subnetwork links “clinical decision support systems” (CDSS) with “acceptance”, highlighting research attention to adoption-related factors within clinical practice. Another cluster connects health informatics, IT, and epidemiology, indicating that AI adoption research extends beyond direct clinical applications to include public health and administrative contexts. Overall, the thematic structure illustrates the breadth of topics addressed in the literature, spanning technical approaches, ethical considerations, adoption dynamics, and system-level applications of AI in healthcare.

The thematic map illustrates a set of recurring thematic areas identified within the literature, reflecting the evolving discourse on AI adoption in healthcare. The analysis of keyword clustering and interconnections highlights patterns related to technological developments, ethical considerations, and application contexts, which inform both theoretical discussions and practical implications. One of the most prominent themes centers on AI and ML, positioned at the core of the network, indicating their centrality within the research corpus. Studies within this cluster examine how ML algorithms are applied to predictive analytics, diagnostic support, and treatment optimization [37]. This thematic area also includes deep learning (DL) applications, reflecting research attention to advanced neural network approaches for processing complex medical data and supporting clinical decision processes [38, 39]. Closely related is the theme of ethics and bioethics, which appears with increasing frequency in recent publications. Research in this cluster addresses issues such as data privacy, algorithmic bias, and decision autonomy in AI-enabled medical systems [40, 41]. This thematic area also encompasses studies on ChatGPT and chatbots, which are examined in relation to clinical documentation, patient interaction, and automated consultations, alongside associated ethical and regulatory considerations [42]. Another major thematic area concerns NLP and medical informatics, which emerge as distinct but interconnected clusters. Studies in this stream explore the integration of NLP models into clinical workflows, including applications such as medical transcription, decision support, and patient monitoring [43–45]. Links with electronic health records (EHRs) are also evident, reflecting research attention to interoperability, data accessibility, and standardization in data-driven healthcare management [46]. Beyond core AI applications, a separate subnetwork relates to clinical decision support systems (CDSS) and acceptance, indicating sustained scholarly attention to adoption-related challenges and implementation barriers [47]. Studies in this area examine factors such as organizational resistance, training needs, and concerns regarding system reliability, which influence the integration of AI-based tools into routine clinical practice [48]. This stream of research focuses on aligning decision-support technologies with clinical workflows and user requirements [49]. Finally, a distinct cluster encompasses health informatics, IT, and epidemiology, highlighting AI-related research extending beyond individual patient care. This literature addresses applications in public health, including disease surveillance, predictive modeling, and administrative decision-making within healthcare organizations [50].

## Discussion

AI adoption in healthcare does not follow a linear trajectory, but rather reflects a multidimensional process in which adoption is contingent upon technological maturity, ethical considerations, and institutional preparedness. Through bibliometric and qualitative content analysis, three recurrent research trajectories can be identified, each representing interrelated but analytically distinct areas of inquiry. These trajectories concern AI-enabled decision support and predictive analytics, ethical and bioethical considerations, and NLP-based AI applications in clinical data management. Together, these themes capture the main areas around which the literature on AI adoption in healthcare is organized. Each trajectory is associated with ongoing scientific debates, reported barriers, and implementation challenges, reflecting the complexity of integrating AI technologies into healthcare systems **(**Table 4**).**

**Table 4:** *Illustrative studies across key AI adoption trajectories in healthcare (TOE-informed classification)*

Trajectory	Article	Title	Citations	Normalised Citations
AI-driven decision support and predictive analysis	Yang et al., (2022)	Unbox the black-box for the medical explainable AI via multi-modal and multi-centre data fusion: A mini-review, two showcases and beyond	402	6,64
	Strohm et al., (2020)	Implementation of (AI) applications in radiology: hindering and facilitating factors	157	1,44
	Zhang et al. (2018)	Towards improving diagnosis of skin diseases by combining deep neural network and human knowledge	107	2,90
	Henry et al. (2022)	Human–machine teaming is key to AI adoption: clinicians’ experiences with a deployed ML system	91	1,5
	Yang J. et al. (2022)	AI healthcare service resources adoption by medical institutions based on TOE framework	30	0,49
Ethical and Bioethical Considerations in AI Implementation	Ye et al. (2019)	Psychosocial Factors Affecting AI Adoption in Health Care in China: Cross-Sectional Study	73	1,35
	Fernandez-Luque et al. (2019)	Humanitarian health computing using AI and social media: A narrative literature review	57	0,6
	Tsopra et al., (2021)	A framework for validating AI in precision medicine: considerations from the European ITFoC consortium	53	1,89
	Maassen et al. (2021)	Future Medical AI Application Requirements and Expectations of Physicians in German University Hospitals: Web-Based Survey	44	1,57
	Bigman et al., (2021)	Threat of racial and economic inequality increases preference for algorithm decision-making.	32	1,14
NLP and AI in Clinical Data Management	Deniz-Garcia et al., (2023)	Quality, Usability, and Effectiveness of mHealth Apps and the Role of AI: Current Scenario and Challenges	40	3,33
	El- Sappagh et al., (2019)	Mobile Health Technologies for Diabetes Mellitus: Current State and Future Challenges	450	3,88
	Park et al. (2024)	Assessing the research landscape and clinical utility of LLMS: a scoping review	29	5,83
	Ahmed et al. (2023)	Harnessing Big Data Analytics for Healthcare: A Comprehensive Review of Frameworks, Implications, Applications, and Impacts	29	2,41
	Esmaeilzadeh P. (2024)	Challenges and strategies for wide-scale AI deployment in healthcare practices: A perspective for healthcare organizations	26	5,2

### AI-driven decision support and predictive analytics

At the heart of AI’s clinical application lies the use of computational models to support complex clinical decision-making, enabling the identification of patterns in large and heterogeneous datasets that may be difficult to detect through human analysis alone. This trajectory examines how ML and DL models are used to support clinicians in diagnostic processes, risk prediction, and treatment planning. At the same time, the literature consistently highlights challenges related to model transparency, clinician trust, and applicability in real-world clinical settings. Yang G. et al. [51] address the black-box problem by proposing multi-modal and multi-center data fusion approaches aimed at improving the interpretability of AI systems. Their work emphasizes that explainability is a key condition for clinical acceptance, particularly in high-stakes decision contexts. Similarly, Strohm et al. [52] document workflow-related challenges in AI-enabled radiology, showing that when AI tools are poorly integrated into existing clinical routines, they may disrupt rather than support practice. Zhang et al. [53] further contribute to this discussion by proposing hybrid human–AI models, in which clinical expertise and DL systems operate in a complementary manner. Their findings suggest that AI effectiveness is closely linked to augmentation rather than full automation, a theme also explored by Henry et al. [54] in their analysis of human–machine teaming in clinical environments. These studies highlight that trust in AI systems develops progressively, through iterative use, validation processes, and active clinician involvement.

This trajectory is further extended by Yang J. et al. [51], who apply the TOE framework to examine organizational readiness for AI adoption. Their findings indicate that AI implementation requires organizational adaptation and coordination, rather than simple technological deployment. Factors such as regulatory constraints, operational workflows, and institutional capabilities play a central role in shaping adoption outcomes. Collectively, this body of research suggests that the effective use of AI in decision support depends on interpretability, integration into clinical practice, and sustained trust-building processes, rather than on technological performance alone.

### Ethical and bioethical considerations in AI implementation

Ethical considerations play a central role in shaping the adoption of AI in healthcare, particularly in relation to algorithmic governance, fairness, and regulatory oversight. This research trajectory examines how ethical concerns influence both organizational decisions and professional acceptance of AI systems. A recurring issue in the literature concerns the tension between the potential benefits of AI-enabled decision-making and concerns related to transparency, accountability, and trust in algorithmic outputs. Ye et al. [55] explore psychosocial responses to AI in healthcare, documenting divergent perceptions among healthcare professionals. While some studies report expectations of efficiency gains, others highlight concerns related to job displacement, loss of professional autonomy, and opaque decision-making processes. These findings suggest that AI adoption involves not only technical change but also cultural and ethical adjustments within clinical environments, with implications for physician–patient relationships. Fernandez-Luque et al. [56] extend this discussion to humanitarian health computing, showing that AI systems developed in high-resource settings may face limitations when applied in low-resource healthcare contexts. Their analysis highlights risks related to data representativeness and contextual mismatch, which may inadvertently exacerbate existing health inequalities if not adequately addressed. Tsopra et al. [57] propose a validation framework for AI in precision medicine, emphasizing that statistical performance alone is insufficient for clinical adoption. Instead, transparency, clinical relevance, and ethical accountability emerge as key criteria. Similarly, Maassen et al. [58] examine physicians’ expectations regarding AI use, identifying algorithmic fairness, bias mitigation, and clarity around responsibility and liability as critical conditions for acceptance. This trajectory is further informed by Bigman et al. [59], who investigate the relationship between AI adoption and socio-economic inequalities. Their findings indicate that perceived bias in algorithmic decision-making, particularly in relation to race and economic status, is associated with increased resistance to AI systems. Collectively, these studies suggest that ethical safeguards are not peripheral considerations but central determinants of sustainable AI adoption in healthcare, influencing legitimacy, trust, and long-term implementation outcomes.

### NLP and AI in clinical data management

Beyond decision-making, AI is increasingly being applied to the processing, retrieval, and structuring of clinical information within healthcare systems. This trajectory examines the integration of NLP and AI-driven automation in clinical data management, including applications related to electronic health records (EHRs), medical transcription, and decision-support tools. While the literature highlights substantial potential benefits, this domain is also associated with challenges related to interpretability, linguistic variability, data security, and regulatory compliance.

Deniz-Garcia et al. [60] examine AI-enabled mobile health applications, showing how NLP techniques are used to support patient engagement and improve the efficiency of clinical documentation. At the same time, their study draws attention to limitations of language-based AI systems, particularly the risk of misinterpretation in clinical contexts. El-Sappagh et al. [61] focus on AI-driven mobile health technologies for diabetes management, illustrating how NLP-based systems can support real-time clinical recommendations. Their findings also point to potential risks associated with over-reliance on automated outputs, highlighting the need for appropriate clinical oversight.

Park et al. [44] extend this line of inquiry to large language models (LLMs), presenting a scoping review of ChatGPT-like systems in clinical settings. Their analysis identifies concerns related to inaccurate or non-contextualized outputs, limited clinical grounding, and variability in system performance, which pose challenges for clinical reliability. Ahmed et al. [62] explore the use of big data analytics in clinical decision support, emphasizing the potential of AI to integrate heterogeneous patient data while also raising issues related to privacy protection, interoperability, and governance. This trajectory is further developed by Esmaeilzadeh [50], who discusses organizational strategies for AI-enabled clinical data standardization. Their work suggests that successful implementation depends not only on technical capabilities but also on regulatory alignment, clinician engagement, and validation processes. Overall, the literature indicates that AI applications in clinical data management require careful balancing between automation and human oversight, particularly given the sensitivity and complexity of healthcare data.

### Main identified adoption obstacles and challenges

Despite the potential of AI to support innovation in healthcare, analysis of the literature through the TOE framework reveals a complex set of interrelated barriers and enabling conditions that shape adoption outcomes [40].

From a technological perspective, AI implementation is frequently associated with challenges related to transparency, explainability, and system integration. The need for interpretable models and compatibility with heterogeneous and often fragmented IT infrastructures complicates deployment within healthcare organizations [43]. Interoperability issues with legacy systems and evolving regulatory requirements are recurrently reported, limiting the practical scalability of AI solutions.

At the organizational level, the literature highlights the importance of cultural readiness and change management. Studies indicate that without organizational cultures supportive of experimentation and digital innovation, AI initiatives may encounter resistance linked to established routines, professional norms, and concerns over workflow disruption [42]. A lack of structured training and limited engagement of clinical staff are commonly identified as factors slowing adoption.

Within the environmental dimension, AI adoption is influenced by the interaction between regulatory constraints and policy-driven incentives. Regulatory frameworks and ethical requirements impose conditions on AI deployment, while public funding programs and national AI strategies provide support mechanisms [50]. However, several studies report persistent gaps in large-scale, multi-site validation of AI systems, which contribute to uncertainty regarding generalizability and clinical reliability [44, 63].

Economic considerations introduce an additional layer of complexity. High implementation and maintenance costs, combined with legal and liability uncertainties, are frequently cited as barriers, particularly in resource-constrained healthcare settings [39]. These factors can limit organizational willingness to invest in AI technologies, even where potential benefits are recognized.

A further barrier identified in the literature concerns the limited knowledge alignment between technical specialists and healthcare professionals. Recent empirical evidence in health services settings shows that unclear role boundaries, limited AI literacy among clinicians, and insufficient cross-professional collaboration significantly hinder AI implementation and contribute to resistance at both individual and organizational levels [64]. While data scientists and AI developers possess advanced technical expertise, clinicians may face difficulties in interpreting AI outputs and underlying methodologies [54]. This gap can affect perceptions of usability and usefulness, increase reliance on intermediaries, and contribute to resistance during early stages of adoption [65]. The literature suggests that insufficient knowledge exchange between these professional groups may hinder trust-building and slow organizational readiness.

Overall, the reviewed studies indicate that addressing AI adoption barriers requires coordinated technological, organizational, and environmental interventions. Multidisciplinary collaboration, iterative validation, and alignment between technical development and clinical practice are consistently highlighted as enabling conditions for effective implementation [52, 66]. Table 5 summarizes the main technological, organizational, and environmental factors identified in the literature, along with the associated barriers and facilitating elements influencing AI adoption in healthcare.

**Table 5:** Summary of AI adoption in healthcare using the TOE framework

Technology	- Use of AI-based decision support systems (DSS), ML, and NLP for clinical decision-making. - Need for explainability in AI systems (e.g., XAI techniques such as SHAP and LIME) to enhance clinician trust. - Integration challenges with existing hospital IT infrastructure and interoperability concerns. - Ethical AI deployment, federated learning for privacy, and bias mitigation strategies. - Performance improvements through advanced DL architectures (e.g., CNNs, RNNs) in medical imaging and diagnostics.
Organization	- Resistance among healthcare professionals due to concerns over loss of autonomy and workflow disruptions. - Need for structured training programs and continuous education on AI applications in clinical practice. - Importance of leadership support and a culture of innovation for successful AI adoption. - Challenges in securing funding and resources for AI implementation and maintenance. - The role of multidisciplinary collaboration among clinicians, data scientists, and regulatory bodies.
Environment	- Influence of regulatory frameworks such as GDPR, HIPAA, and national AI policies on adoption rates. - Ethical concerns surrounding AI-driven clinical decisions and their potential impact on patient trust. - Variability in AI readiness across different healthcare institutions and geographic regions. - Need for standardized evaluation models and structured policy guidelines to ensure safe AI deployment. - Disparities in access to AI technologies between high-resource and low-resource healthcare settings.
Barriers	- Limited transparency and interpretability of AI models, leading to trust issues among clinicians. - High implementation costs and the financial burden of AI integration into healthcare systems. - Fragmentation of research and lack of standardized protocols for AI deployment. - Insufficient real-world validation of AI models, leading to skepticism about clinical efficacy. - Interoperability challenges with electronic health records (EHRs) and existing clinical workflows.
Facilitators	- Increased efficiency in clinical workflows and reduced cognitive workload for healthcare professionals. - AI-driven improvements in diagnostic accuracy and personalized treatment recommendations. - Government incentives and policy support for AI research and implementation. - Emerging frameworks for ethical AI, ensuring transparency, fairness, and accountability. - Patient education initiatives to improve public trust in AI-driven healthcare solutions

### Focus on knowledge-proximity between IT scientists and physicians

**Fig. 9:**
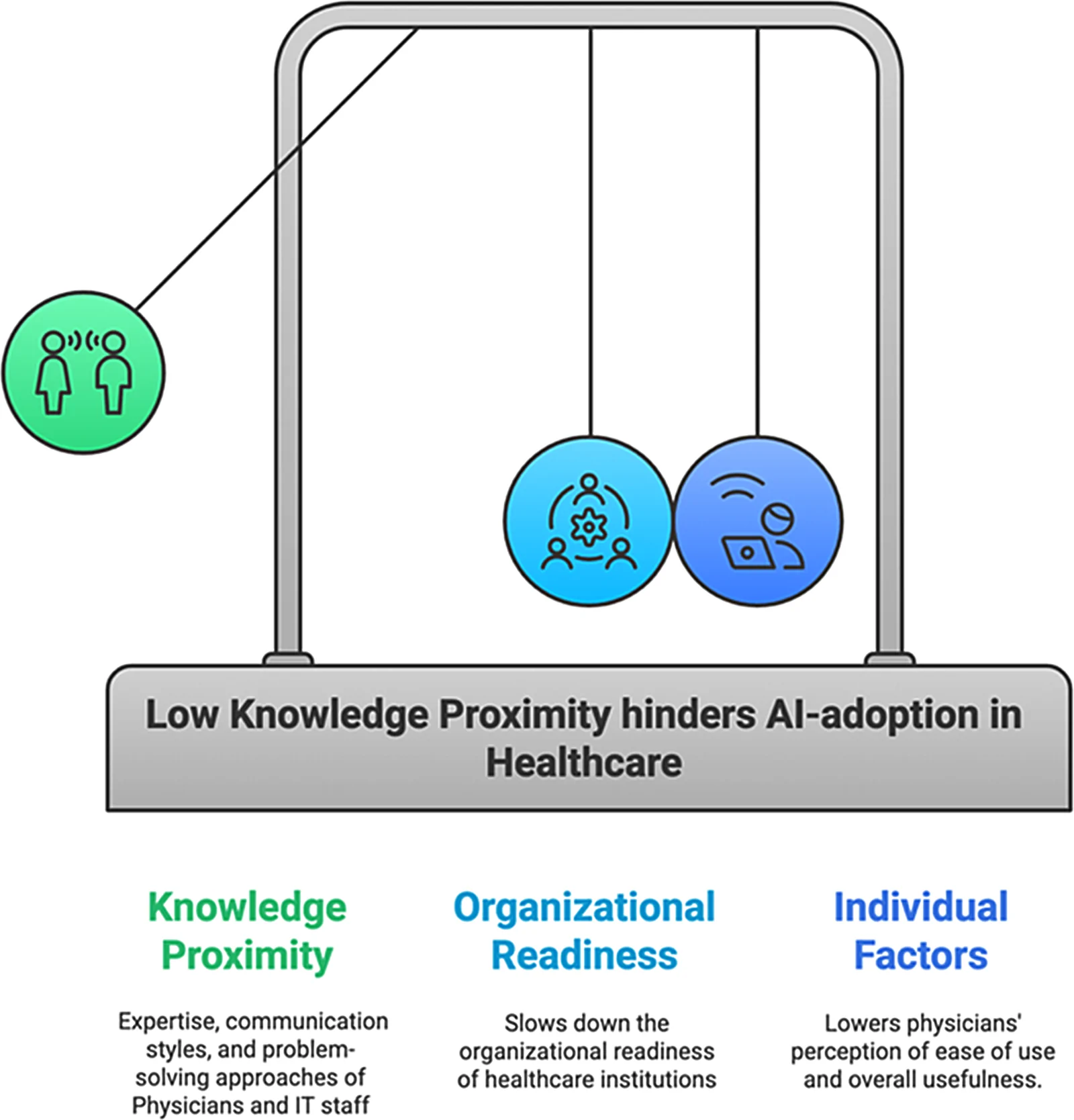
Conceptual illustration of knowledge-alignment challenges influencing AI adoption in healthcare

Figure 9 summarizes the main integrative insight emerging from this review, illustrating how limited knowledge alignment between physicians and IT specialists is recurrently identified in the literature as a barrier to AI adoption in healthcare. The figure uses a conceptual representation to show how misalignment between clinical and technical expertise can generate cascading challenges across organizational and individual dimensions, affecting the implementation and use of AI systems.

Within the TOE framework, this form of knowledge misalignment is associated with lower organizational readiness, contributing to delays in implementation and increased friction during system deployment. Physicians, while possessing deep clinical expertise, may have limited familiarity with the technical principles underlying AI-based tools, which can foster skepticism regarding their applicability and reliability. Conversely, IT specialists, although proficient in algorithm development and system integration, may lack contextual understanding of clinical workflows, ethical considerations, and decision-making practices. The literature suggests that this disconnect can result in AI solutions that are insufficiently aligned with clinical needs, limiting their effective use within healthcare organizations.

From an individual-level perspective, insights from the TAM help explain how perceptions of usefulness and ease of use are shaped by this knowledge gap. Studies indicate that clinicians may experience difficulties in interpreting or trusting AI-generated recommendations, particularly when systems lack transparency or intuitive interfaces. This challenge is further amplified when clinical expertise is weakly integrated into data curation and model development processes, increasing the risk of biased or contextually inappropriate outputs. As a result, limited involvement of clinicians in these stages may undermine confidence in AI tools and reduce willingness to adopt them. The figure also highlights how these dynamics may reinforce one another over time, as low engagement with AI systems constrains opportunities for iterative refinement and contextual adaptation. In high-pressure clinical environments, tools perceived as opaque or poorly integrated into workflows are less likely to be adopted, further slowing learning and improvement processes.

Overall, Fig. 9 emphasizes that AI adoption in healthcare cannot be understood through isolated organizational or individual lenses alone. Rather, the literature points to the need for coordinated consideration of organizational readiness (TOE) and user acceptance factors (TAM). Organizational structures that support AI implementation may be insufficient if clinicians remain unable or unwilling to integrate AI into practice, while technically well-designed systems may fail without supportive institutional conditions. Accordingly, the reviewed studies consistently highlight the importance of interdisciplinary collaboration and continuous knowledge exchange between clinical and technical actors. Active involvement of clinicians in system design, data preparation, and validation processes emerges as a key condition for aligning AI tools with clinical practice and supporting sustainable adoption.

This alignment extends beyond technical training or medical literacy, referring instead to the development of shared understanding and communication between clinical and technical actors, which supports the design of AI solutions that are both clinically relevant and technically feasible. The literature suggests that limited alignment may result in siloed development processes, leading to systems that are poorly integrated into clinical workflows or, conversely, to clinicians who experience difficulties in trusting and effectively using AI tools. Accordingly, early knowledge alignment emerges as a recurrent enabling condition for AI adoption, rather than as a purely technical prerequisite.

Once this gap is addressed, studies report improvements in cross-professional collaboration. Enhanced communication between IT developers and medical professionals is associated with more effective problem-solving, iterative system refinement, and smoother implementation processes. When both groups are actively involved in AI development and deployment, barriers related to technical complexity and clinical skepticism are reported to decrease, and AI tools are more often perceived as jointly developed resources rather than externally imposed technologies.

As collaboration strengthens, organizational readiness may increase, reflecting changes in institutional structures, routines, and decision-making processes. In this phase, AI is increasingly discussed in strategic terms rather than as an experimental add-on. Alignment between technological capabilities and clinical needs is associated with fewer implementation frictions, including reduced resistance at leadership levels and fewer operational disruptions during deployment.

At the individual level, insights from the TAM help explain how these organizational conditions influence user perceptions. When clinicians gain familiarity and confidence with AI tools, perceived usefulness and ease of use tend to improve, supporting acceptance. Similarly, IT specialists benefit from higher likelihoods of system uptake, reducing inefficiencies associated with low adoption. The literature indicates that increased trust and familiarity can mitigate psychological and operational barriers that initially slow adoption.

**Fig. 10:**
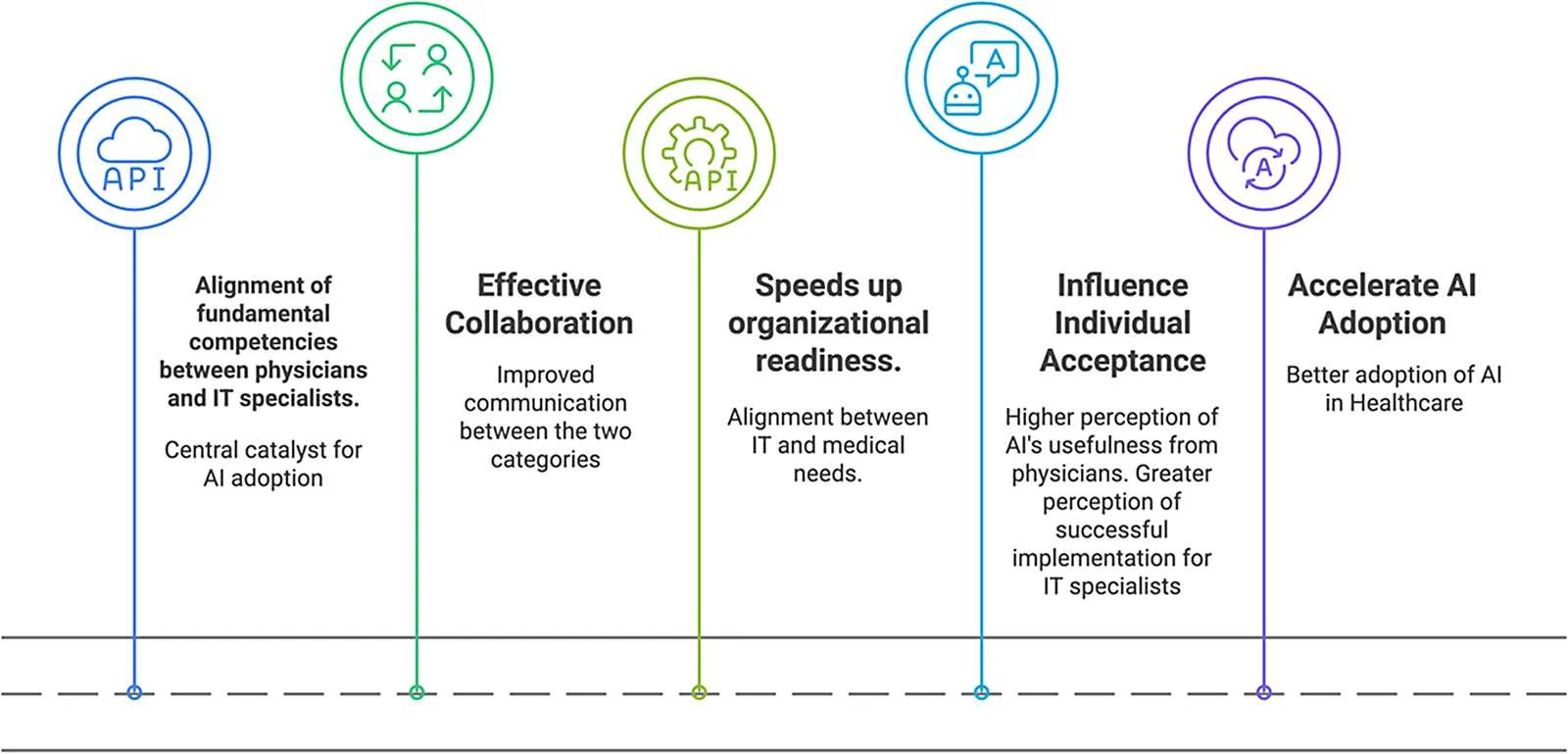
Conceptual illustration of knowledge alignment processes supporting AI adoption in healthcare

The sequence illustrated in Fig. 10 conceptualizes AI adoption as a multi-level process, shaped by interactions between knowledge alignment, collaboration, organizational readiness, and individual acceptance. Rather than representing a linear pathway or guaranteed outcome, the figure synthesizes evidence suggesting that early attention to knowledge alignment can facilitate more coordinated and sustainable AI implementation processes. In this sense, AI adoption in healthcare is framed as a socio-technical transformation requiring interdisciplinary engagement from the outset, rather than as a standalone technical intervention.

Building on the patterns identified in the reviewed literature, Knowledge-proximity in AI adoption in healthcare refers to the degree of cognitive and operational alignment between professional groups involved in AI implementation. It encompasses shared interpretive frames, overlapping domains of expertise, and reciprocal understanding of task-specific constraints. In healthcare settings, limited Knowledge-proximity often reflects structured epistemic asymmetries between clinicians and IT specialists, rooted in differences in professional training, evaluative criteria, and domain-specific language. These asymmetries can hinder the translation of algorithmic outputs into clinically meaningful action, thereby conditioning both implementation processes and patterns of technology acceptance. Unlike generic communication gaps, Knowledge-proximity captures enduring epistemic distance that systematically shapes how AI tools are interpreted, trusted, and operationalized within clinical workflows. When this gap is reduced, studies report improvements not only in communication, but also in trust, problem-solving processes, and organizational adaptability.

From the perspective of the TAM, Knowledge-proximity helps interpret variation in perceived ease of use and perceived usefulness observed in empirical studies. When clinicians possess a basic understanding of AI principles, or when IT specialists develop greater awareness of clinical workflows, the literature suggests a reduction in cognitive burden associated with interacting with AI systems. In such cases, opaque machine-learning outputs may be perceived as more interpretable and reliable, supporting more favorable user perceptions and higher acceptance. Knowledge alignment does not eliminate professional boundaries; rather, it reduces cognitive friction, allowing clinicians to engage more confidently with AI-supported decision processes.

At the organizational level, the TOE framework situates Knowledge-proximity within broader dynamics of readiness and coordination. AI implementation outcomes are repeatedly shown to depend not only on technological maturity, but on the ability of organizations to synchronize knowledge across professional domains. Healthcare institutions that actively promote interdisciplinary interaction and learning are more likely to create environments supportive of AI experimentation and gradual diffusion. In these contexts, barriers such as miscommunication, resistance to change, and skepticism toward algorithmic systems are reported to be less pronounced, supporting smoother implementation trajectories. Taken together, the integration of TAM and TOE perspectives suggests that Knowledge-proximity operates as a cross-cutting mechanism linking individual acceptance and organizational readiness, rather than as an independent driver of adoption. It does not replace existing adoption theories, but helps explain how micro-level perceptions and macro-level structures interact during AI implementation. By aligning clinical and technical knowledge bases, healthcare organizations may reduce the risk of misaligned system design and underutilization, increasing the likelihood that AI tools remain grounded in clinical practice rather than detached from operational realities.

**Fig. 11:**
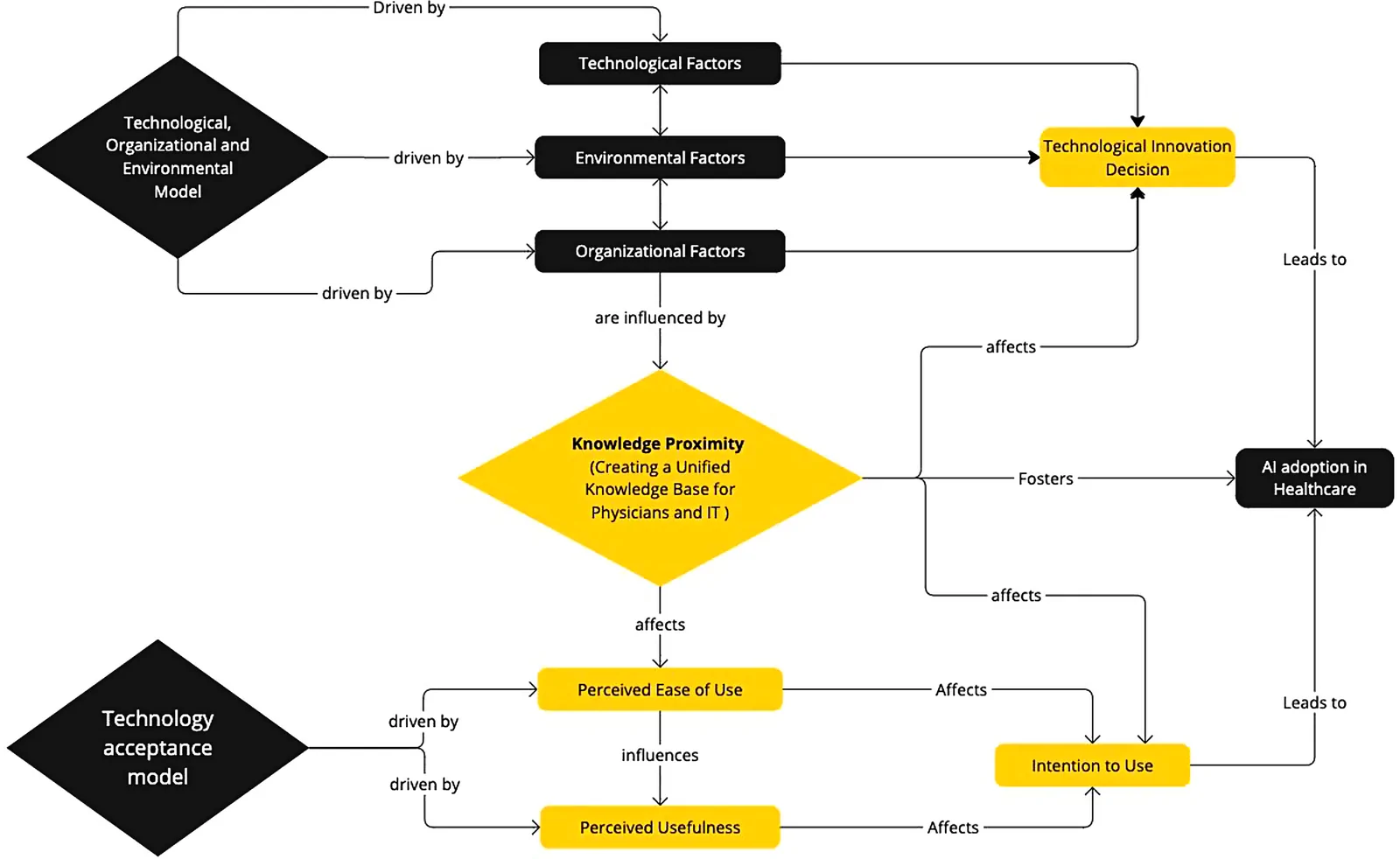
The role of Knowledge-proximity in the process of AI adoption within the healthcare sector

Therefore, Knowledge-proximity seizes a recurring pattern in the literature concerning the alignment of cognitive and operational dimensions of AI adoption. By framing this alignment through established lenses such as TAM and TOE, the concept supports a more integrated interpretation of AI adoption processes in healthcare, clarifying how individual perceptions and organizational structures interact during implementation. Figure 11 synthesizes the cross-level mechanisms discussed in this section and anticipates the contextual patterns examined in Section “Cross-contextual patterns in AI adoption”, integrating individual acceptance dynamics, organizational readiness, and knowledge-alignment processes into a unified interpretive framework.

### Cross-contextual patterns in AI adoption

Although the determinants of AI adoption identified in this review can be organized within technological, organizational, and environmental dimensions, the analyzed studies consistently indicate that their relative salience and interaction patterns vary across healthcare settings. Rather than operating as isolated variables, these determinants appear to form context-dependent configurations shaped by institutional environments, clinical domains, and organizational capabilities. In several contexts, these configurations are further shaped by varying degrees of Knowledge-proximity between clinical and technical actors.

First, institutional and regulatory environments influence implementation trajectories. Several reviewed studies conducted in publicly funded or highly regulated systems highlight how formal validation requirements, governance procedures, and accountability structures condition the pace and structure of AI deployment [42, 52]. In such contexts, adoption processes tend to be incremental and closely tied to institutional oversight. Other studies focusing on implementation within hospital networks and specialized clinical units suggest that organizational priorities, such as efficiency gains or service innovation, shape how experimentation and pilot testing are structured [48, 50]. Collectively, the reviewed evidence indicates that institutional configuration mediates how technological readiness translates into operational adoption.

Second, domain specificity substantially shapes adoption dynamics. A significant portion of the literature examines imaging-based AI applications, where algorithmic outputs can be benchmarked against established diagnostic standards [47, 52]. In these contexts, measurable performance indicators and comparability with human assessment often facilitate perceived usefulness and trust. By contrast, studies addressing clinical decision support systems and predictive analytics emphasize challenges related to interpretability, workflow integration, and professional judgment [38, 54, 64]. Here, adoption depends less on raw predictive performance and more on alignment with clinical routines and decision-making practices. This variation suggests that epistemic alignment and workflow compatibility play a more prominent role in domains where AI outputs are less directly verifiable against standardized benchmarks.

Third, organizational maturity and digital infrastructure moderate implementation processes. Studies adopting TOE-informed perspectives and empirical investigations of implementation contexts indicate that institutions with prior experience in digital health initiatives and interdisciplinary collaboration exhibit higher levels of readiness [14, 37, 48]. This readiness includes established coordination routines, clearer governance structures, and reduced cognitive friction between professional groups. Conversely, resource-constrained settings and institutions with limited digital integration report compounded barriers, where infrastructural gaps reinforce skepticism and limit opportunities for iterative learning [50].

Taken together, these cross-contextual patterns suggest that AI adoption in healthcare operates through interacting mechanisms rather than isolated determinants. Technological performance alone is rarely sufficient to secure implementation. Instead, adoption outcomes depend on how technological attributes, institutional structures, domain characteristics, and professional knowledge alignment converge within specific organizational environments. These contextual variations further substantiate the integrative logic presented in Fig. 11, illustrating how adoption determinants operate differently across institutional and domain-specific settings.

### Practical and policy implications

The cross-contextual patterns identified in Section “Cross-contextual patterns in AI adoption” highlight several actionable implications for health service managers and policy-makers.

First, reducing epistemic distance between clinical and technical professionals requires structured governance mechanisms rather than ad hoc collaboration. The reviewed studies suggest that organizations benefit from institutionalized coordination structures, such as interdisciplinary AI steering committees, the integration of clinical specialists within data science teams, and joint training initiatives focused on AI literacy and workflow integration. These mechanisms can formalize knowledge alignment and reduce misinterpretation of algorithmic outputs during implementation.

Second, implementation strategies should be tailored to the specific AI domain. Evidence from imaging-based applications indicates that performance benchmarking protocols and validation studies embedded within established diagnostic routines facilitate integration. By contrast, studies on clinical decision-support systems emphasize the importance of workflow redesign, iterative usability testing, and explainability mechanisms that enable clinician feedback. A uniform implementation model is therefore unlikely to be effective across heterogeneous AI applications; instead, domain-sensitive deployment pathways appear more consistent with the reviewed evidence.

Third, organizational readiness assessments should extend beyond technological infrastructure to include coordination capacity and interdisciplinary experience. The literature indicates that prior digital health initiatives, established data governance frameworks, and cross-departmental communication structures are associated with smoother implementation trajectories. Incorporating these dimensions into formal readiness audits and funding criteria may enhance the sustainability of AI adoption initiatives.

Finally, regulatory and institutional actors play a central role in shaping trust and legitimacy. Transparent validation standards, clearly defined accountability frameworks, and participatory evaluation processes involving frontline clinicians are repeatedly associated with reduced skepticism and more responsible integration of AI tools within clinical practice.

Overall, these implications suggest that effective AI adoption in healthcare is not solely a technological undertaking but a coordinated organizational and governance process requiring deliberate alignment of knowledge bases, clinical workflows, and institutional incentives.

## Conclusions

Through this literature review, we identify that AI adoption in healthcare extends far beyond a purely technological shift, reflecting a broader reconfiguration of how medical services are organized, delivered, and supported. While AI holds the potential to enhance diagnostics, streamline workflows, and support patient care, its implementation remains uneven and highly context-dependent. Institutional inertia, professional skepticism, fragmented infrastructures, and regulatory uncertainty consistently emerge in the literature as constraints, slowing progress and complicating integration. Overall, the findings confirm that AI adoption is not solely a matter of technological readiness but rather a socio-technical process shaped by institutional arrangements, professional practices, and systemic conditions.

Within this complex landscape, the review highlights the recurrent importance of Knowledge-proximity between IT specialists and healthcare professionals. The literature suggests that the disconnect between these groups represents a persistent barrier influencing the success or failure of AI implementation efforts. When technical developers and clinicians operate in relative isolation, AI solutions are more likely to be perceived as misaligned with clinical workflows, decision-making routines, and frontline operational needs. A lack of shared understanding and interdisciplinary interaction is frequently associated with resistance, limited trust, and underutilization of AI tools, even when technical performance is high.

This review positions Knowledge-proximity as an integrative interpretive mechanism linking insights from established adoption frameworks, including TOE and TAM. While prior research has extensively examined technological, organizational, and environmental determinants of AI adoption, the evidence synthesized here indicates that cognitive and professional distance between developers and users conditions both organizational readiness and individual acceptance. From this perspective, AI adoption depends not only on readiness or capability, but on the alignment of knowledge bases and interpretive frames across professional groups.

From a practical standpoint, the review underscores the importance of institutionalized coordination structures, domain-sensitive implementation strategies, and organizational readiness assessments that extend beyond technological infrastructure. The evidence suggests that interdisciplinary governance mechanisms, structured validation pathways, and participatory evaluation processes can facilitate more sustainable integration of AI into routine care. Effective adoption therefore requires deliberate alignment of knowledge, workflows, and institutional incentives.

This review is subject to several limitations. The analysis was restricted to peer-reviewed journal articles indexed in Scopus and primarily published in high-impact outlets, which, while ensuring methodological consistency, may have excluded emerging or practice-oriented contributions. In addition, as a literature-based synthesis, the study reflects reported patterns rather than primary empirical validation of the proposed integrative framework. The heterogeneity of healthcare contexts and AI applications further limits causal generalization.

Future research could advance this field through comparative empirical studies across healthcare systems, domain-specific investigations of implementation pathways, and quantitative testing of the relationship between knowledge alignment, organizational readiness, and user acceptance. Longitudinal and multi-site designs would be particularly valuable in clarifying how governance structures and interdisciplinary collaboration influence the sustainability of AI integration over time. In conclusion, by foregrounding Knowledge-proximity, this review reframes AI adoption in healthcare as a human-centered and organizationally mediated process rather than a purely technical challenge. The long-term impact of AI will depend on the capacity of healthcare organizations to bridge disciplinary boundaries and institutionalize shared understanding. Where such conditions are cultivated, AI is more likely to be integrated in ways that are clinically meaningful, organizationally sustainable, and aligned with the realities of healthcare practice.

## Supplementary Information

Below is the link to the electronic supplementary material.

Supplementary Material 1

## Data Availability

All data analysed during this study are included in the published articles reviewed. No additional datasets or code were generated.

## Abbreviations

AI: Artificial Intelligence
IT: Information Technology
DL: Deep Learning
ML: Machine Learning
NLP: Natural Language Processing
PRISMA: Preferred Reporting Items for Systematic Reviews and Meta-Analyses
TOE: Technology–Organization–Environment; EHRs: electronic health records
